# Single cell transcriptome sequencing of stimulated and frozen human peripheral blood mononuclear cells

**DOI:** 10.1038/s41597-023-02348-z

**Published:** 2023-07-06

**Authors:** Céline Derbois, Marie-Ange Palomares, Jean-François Deleuze, Eric Cabannes, Eric Bonnet

**Affiliations:** Centre National de Recherche en Génomique Humaine (CNRGH), Institut de Biologie François Jacob, CEA, Université Paris-Saclay, Evry, France

**Keywords:** Gene expression analysis, Genomics

## Abstract

Peripheral blood mononuclear cells (PBMCs) are blood cells that are a critical part of the immune system used to fight off infection, defending our bodies from harmful pathogens. In biomedical research, PBMCs are commonly used to study global immune response to disease outbreak and progression, pathogen infections, for vaccine development and a multitude of other clinical applications. Over the past few years, the revolution in single-cell RNA sequencing (scRNA-seq) has enabled an unbiased quantification of gene expression in thousands of individual cells, which provides a more efficient tool to decipher the immune system in human diseases. In this work, we generate scRNA-seq data from human PBMCs at high sequencing depth (>100,000 reads/cell) for more than 30,000 cells, in resting, stimulated, fresh and frozen conditions. The data generated can be used for benchmarking batch correction and data integration methods, and to study the effect of freezing-thawing cycles on the quality of immune cell populations and their transcriptomic profiles.

## Background & Summary

Peripheral blood mononuclear cells (PBMCs) are blood cells that are a critical part of the immune system used to fight off infection. They work together to protect our bodies from harmful pathogens. Due to their primary location in peripheral blood, they act as a line of defense against infection and disease. PBMCs are used by medical researchers to study immune cell behavior when exposed to various pathogens, disease progression in the human body and factors affecting long-term immunity^1^. PBMCs are used in a multitude of different areas, such as vaccine development, infectious disease study, immunology, disease modeling and biomarker identification, just to name a few. However, due to the complexity of PBMCs, which contain multiple different cell types, studying the function of the individual cell types can be difficult, and studies often rely on bulk measurements. Single-cell RNA-sequencing (scRNA-seq) approaches can be used to overcome these problems, allowing for the identification and quantification of the subpopulation of cells that make up the PBMC sample quite easily. ScRNA-seq has emerged as a central tool for identifying and characterizing cell types, states, lineages and circuitry^2^. The rapid growth in the scale and robustness of laboratory protocols and associated computational tools has opened the way to substantial scientific discoveries. ScRNA-seq was also applied to the sequencing and analysis of PBMCs transcriptomics in various health and disease contexts. For instance this technology was used quite recently to analyze the immune response landscape of COVID-19 and Influenza patients^3^. In another study, Wang and colleagues used scRNA-seq on PBMCs to study the global immune response in Kawasaki disease (KD) patients. KD is the most common cause of acquired heart disease in children in developed countries. The study showed that the most differentially expressed genes were found in monocytes, with high expression of pro-inflammatory mediators, immunoglobulin receptors and low expression of MHC class II genes in acute KD, and that the percentage of CD8+ T cells is decreased in acute KD, and notably effector memory CD8+ T cells compared to healthy controls^4^. Here we use scRNA-seq to sequence more than 30,000 cells from human PBMC samples to high depth of coverage (>100,000 reads/cell). We provide data from resting and stimulated PBMCs. We used lipopolysaccharides (LPS) bacteria extracts to induce an immune response and transcriptional changes. We also provide a dataset where cells have been frozen according to a demonstrated protocol for the preparation of fresh frozen human PBMCs for scRNA-seq analysis. We illustrate how those datasets can be used for benchmarking computational methods for data integration and to analyse the consequences of freezing/thawing cells before scRNA-seq library preparation.

## Methods

### Participant information

Fresh blood was provided by the EFS (Etablissement Francais du Sang) from two healthy anonymous donors (one adult female and one adult male) who gave informed consent for experimental research work. The EFS is a public administrative body responsible for collecting, preparing, qualifying and distributing labile blood products (blood, plasma, platelets) for blood transfusion in France. EFS is authorised, for its non-transfusion activities, to collect, prepare, store and sell blood or its components for teaching or research purposes, excluding any therapeutic use. Only adults (older than 18 years old) can give their blood and harvesting is only carried out with the written consent of the donor. The doctor informs the donor of the importance of biological samples for the progress of medical research.

### PBMCs isolation

**Fig. 1:**
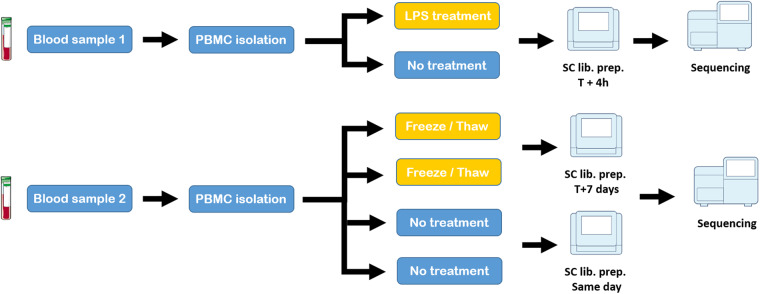
Schematic overview of experimental design. For the LPS vs No treatment study, two samples (technical replicates) were generated from the same blood sample. Single-cell library preparation was performed after 4 hours of treatment/no treatment, followed by sequencing. For the Frozen vs no treatment study, four samples (technical replicates) were generated from the same blood sample. Two replicates were frozen for 7 days, thawed (following the manufacturer demonstrated protocol for human frozen PBMCs), and processed for single-cell libraries preparation. The last two replicates were processed for single-cell libraries preparation immediately after isolation. Note that frozen and fresh libraries were prepared on different days (but on the same machine). All four fresh/frozen libraries were sequenced on the same machine. Note that the sample used for the LPS experiment corresponds to the adult male anonymous donor, while the sample used for the fresh/frozen experiments corresponds to the adult female anonymous donor.

Twelve ml (2 tubes by donor) of fresh blood was collected in EDTA anticoagulant. PBMCs were isolated using HISTOPAQUE-1077 (Sigma Aldrich; cat# 10771-6X100ML). After centrifugation (285 rcf × g for 5 min at room temperature without the brake), PBMCs remained at the plasma-HISTOPAQUE-1077 interface and were carefully transferred to a new tube. PBMCs were washed two or three times with 3x volume 1X PBS (cat# 10010023 Gibco) followed by a centrifugation (285 rcf × g for 5 min at room temperature without the brake). A visual check was done after each centrifugation to ensure that no platelets remain. After that, we followed two different paths (Fig. 1). (1) PBMCs were re-suspended until uniform cell suspensions were obtained in 10 mL of culture medium: RPMI 1640 Medium, GlutaMAXTM supplement (cat# 61870044 Gibco) with 10% FBS (Fetal bovine serum cat# 10270106 Gibco) and 2% Pennicilline/streptomycine (cat# 15070063 Gibco). The PBMCs were then incubated overnight at 37 °C, 5% CO2. For the stimulated sample we used LPS 1 µg/1 mL (Lipopolysaccharides from Escherichia coli O111:B4; cat# L5293-2ML Sigma Aldrich) incubated at 37 °C for 4 hours. LPS is used to induce inflammatory response and transcriptomic changes. The sample with no treatment (the “resting” sample) was incubated at 37 °C for 4 hours. After incubation, PBMCs were used for the cell suspension preparation step. (2) PBMCs were frozen according to the manufacturer’s protocol (Fresh Frozen Human Peripheral Blood Mononuclear Cells for Single Cell RNA sequencing CG00039 revD, 10X Genomics). The cells were stored in a −80 °C freezer for at least one week and thawed according to the protocol before cell suspension preparation. For the samples that were not frozen, PBMCs after resuspension were used directly for the cell suspension preparation. Note that the sample used for the LPS experiment corresponds to the adult male anonymous donor, while the sample used for the fresh/frozen experiments corresponds to the adult female anonymous donor.

### Cell suspension preparation

PBMCs were counted with a Malassez counting chamber, dead cells rate and cellular debris were checked. The cells were centrifuged at 285 rcf × g for 5 min at room temperature without the brake. The supernatant was removed, and the cell pellet was resuspended in 1 mL 1X PBS (cat# 10010023 Gibco) containing 0.04% filtered Bovine Serum Albumin (BSA, 50 mg/ml cat#AM2616, ThermoFischer Scientific). Resuspended cells were centrifuged at 285 rcf × g for 5 min at room temperature. The supernatant was removed delicately and the cell pellet was resuspended in 1 mL 1X PBS containing 0.04% filtered BSA. For the frozen cells, PBMCs were thawed and resuspended according to the manufacturer’s protocol (Fresh Frozen Human Peripheral Blood Mononuclear Cells for Single Cell RNA sequencing CG00039 revD, 10X Genomics). In the end, the cells are also resuspended in 1 ml 1X PBS containing 0.04% filtered BSA. Cells were again counted with a Malassez counting chamber. According to the manufacturer’s protocol, a concentration between 700,000 and 1,200,000 cells per ml is required for the next step. Consequently the volume was adapted if needed to reach this concentration range.

### Single cell library preparation and sequencing

Single Cell RNA-Seq was performed using the 10X Genomics Chromium Next Gem Single cell 3′ Reagents Kits v3.1 (PN-1000121, CG000315 protocol rev A, 10X Genomics) and Dual Index kit TT set A (PN- 1000215, 10X Genomics) according to the manufacturer’s instructions. For the resting and stimulated samples, the target was estimated at 8000 cells, while for the fresh and frozen samples, the target was set at 6,000 cells. Briefly, the cell suspension barcoded gel beads and partitioning oil were loaded onto the 10X Genomics Chromium Chip (Next GEM chip G) to generate single cell Gel Beads In emulsion (GEMs). Captured cells were lysed and the transcripts were barcoded through reverse transcription inside individual GEMs. The constructed libraries were sequenced on Illumina NextSeq500 (FC High output 300 cycles) or Novaseq (FC S1 standard 200 cycles) and processed using Cell Ranger version 6.0.1 (10X Genomics).

### Data processing and analysis

The fastq and index files were processed with Cell Ranger and the human genome GRCh38 as the reference (10X Genomics annotation file refdata-gex-GRCh38-2020-A). The R package Seurat^5^ version 4.1.0 was used for quality control and most of the analysis. In addition, we used Scrublet^6^ for doublets estimation and the software tool MultiMAP^7^ for certain steps of dimensionality reduction. In order to annotate the clusters described on Fig. 6, we used the Seurat functions “FindTransferAnchors” and “TransferData” to transfer cell type labels from a reference dataset onto a new query dataset. The reference dataset in this case is a Human PBMC reference dataset included in the package Seurat (dataset “ifnb”). The complete procedure for data transfer, as well as all the procedures used to produce the figures and analyses showed in this study, are available on our GitHub repository (https://github.com/erbon7/sc_pbmc).

## Data Records

The raw data consisting of nucleotide sequences (FASTQ files) along with Cell Ranger filtered feature-barcode matrices (MEX format, file barcodes.tsv.gz, features.tsv.gz, matrix.mtx.gz) for each sample are publicly available in the NCBI GEO database (GEO:GSE226488)^8^.

## Technical Validation

After sequencing, all the samples were processed with Cell Ranger with default parameters for quality control and gene counting. For the resting and stimulated samples, we set the “–expect-cells” parameter to 8,000 and for the fresh and frozen samples we set this parameter to 6,000. We used the human genome GRCh38 as the reference (10X Genomics annotation file refdata-gex-GRCh38-2020-A). Doublets rates were estimated with the software Scrublet^6^, using the default parameters. The Cell Ranger summary reports, which include many different quality indicators, did not report any value outside the expected ranges. Table 1 reports the indicators for the LPS treatment experiment. For comparison, we also included in this table the indicators obtained from a similar dataset publicly available from 10X Genomics that we use here as a reference^9^. This PBMC dataset comes from a healthy donor and was sequenced at a depth of approximately 110,000 reads/cell, for a target of 10,000 cells. The FASTQ files were downloaded from the site and processed with the same version of Cell Ranger and with the same parameters. We can see in Table 1 that the quality indicator values for the resting and stimulated PBMCs are very similar to the values for the reference 10X Genomics sample. We have a median number of genes per cell around 2,300 and a mean number of reads per cell of 115,000 for the resting and stimulated samples, thus achieving a sequencing saturation of 79 and 76% respectively, slightly better than the 10X Genomics reference dataset. For the percentage of reads mapped to the genome and the transcriptome, the values are almost identical. The rate of doublets, estimated with the Scrublet software on the raw counting matrices, is within expected values and is slightly lower for the resting and stimulated datasets compared to the 10X Genomics reference set. The median number of mitochondrial genes, a common indicator of apoptotic cells, is well below the commonly used threshold of 10% for both the resting and the stimulated datasets. For the frozen/non frozen PBMC samples, there are four samples in total (two technical replicates for the fresh conditions and two technical replicates for the frozen conditions). All the indicators are shown in Table 2, and are within the acceptable range. There was no special warning in the Cell ranger report. For the fresh samples, the values are similar to the values obtained for the resting PBMC experiment. The median number of genes per cell for the fresh samples is around 2,100 with a mean number of reads per cell of 100,000 and a doublet rate of 7–8%. For the frozen samples, the mean number of genes per samples is lower, around 1,600 genes per cell, with a slightly higher mean number of reads per sample and a lower doublets rate of 3–5%. The sequencing saturation is high for all samples, with values that are between 77 and 85%. For all the samples we have a median number of mitochondrial genes below the usual threshold of 10%. Taken together, all these results demonstrate the high quality of our human PBMC scRNA-seq datasets.

**Table 1:** Quality control indicators for the resting and stimulated PBMC samples.

	10X Gen.	PBMC resting	PBMC stimulated
Target nb of cells	10,000	8,000	8,000
Estimated nb of cells	10,915	6,876	7,895
Median nb of genes per cell	2,310	2,322	2,487
Mean nb of reads per cell	110,728	115,573	114,721
Fraction reads in cells	95%	95.5%	95.4%
Total genes detected	25,638	25,791	26,012
Median UMI counts per cell	8,504	8,488	10,129
Total nb of reads	1.2 B	788 M	901 M
Sequencing saturation	71%	79%	76%
Reads mapped to genome	96%	96%	96%
Reads mapped to transcriptome	56%	54%	54%
Median nb of mitochondrial genes	6.5%	5.8%	5.1%
Doublets rate	8.3%	7.3%	6.7%
Valid barcodes	98.3%	96.7%	96.8%
Valid UMIs	99.9%	100%	100%
Q30 bases in barcode	97%	93.8%	94%
Q30 bases in RNA read	95.4%	92.2%	91.8%
Q30 bases in UMI	96.9%	93.4%	93.6%

10X Gen.: 10X Genomics public PBMC sample.

**Table 2:** Quality control indicators for the fresh and frozen PBMC samples.

	PBMC fresh 1	PBMC fresh 2	PBMC frozen 1	PBMC frozen 2
Target nb of cells	6,000	6,000	6,000	6,000
Estimated nb of cells	3,942	4,336	3,820	3,920
Median nb of genes per cell	2,094	2,030	1,590	1,664
Mean nb of reads per cell	105,465	95,118	121,573	121,545
Fraction reads in cells	93.5%	93.2%	73.7%	81.7%
Total genes detected	23,703	23,864	23,438	23,267
Median UMI counts per cell	8,079	7,852	5,555	6,385
Total nb of reads	416 M	412 M	465 M	476 M
Sequencing saturation	80%	77%	82%	85%
Reads mapped to genome	95%	91%	93%	94%
Reads mapped to transcriptome	53%	51%	39%	45%
Median nb of mitochondrial genes	6.2%	6.1%	5%	4.5%
Doublets rate	6.8%	8.9%	4.7%	3.3%
Valid barcodes	97.3%	97.3%	96.6%	96.7%
Valid UMIs	99.9%	99.9%	99.9%	99.9%
Q30 bases in barcode	95.1%	95.2%	95%	94.6%
Q30 bases in RNA read	93.3%	93.7%	93.1%	92.6%
Q30 bases in UMI	94.8%	94.9%	94.7%	94.3%

## Usage Notes

### Resting versus stimulated PBMC

For this analysis example, we filtered the two PBMC datasets to remove low quality cells. First we removed the doublets predicted by Scrublet (308 for resting PBMCs and 344 cells for stimulated). Then, we analysed the distribution of the median number of genes per cell and the percentage of mitochondrial genes per cell to define the filtration thresholds. We removed cells having a low or very high median number of genes (>200 and <7000 for resting, >200 and <6200 for stimulated PBMCs), and cells having a percentage of mitochondrial genes greater than 14%. After filtering, we were left with 6,206 cells and 7,057 cells for resting and stimulated PBMCs respectively.

**Fig. 2:**
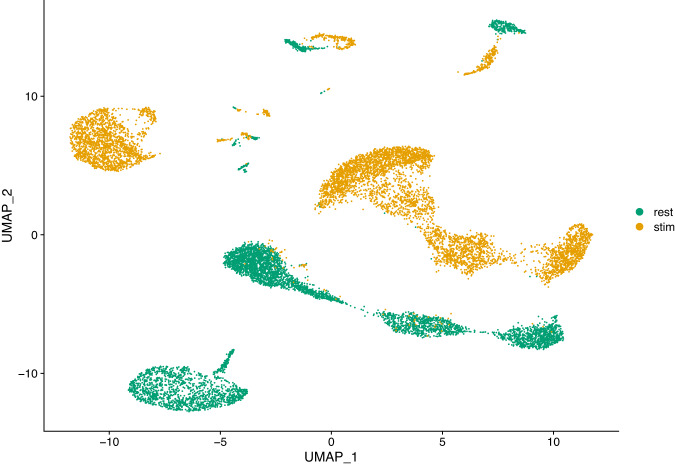
UMAP plot of scRNA-seq data for the resting versus stimulated PBMC cells. rest = resting PBMCs, stim = stimulated PBMCs.

**Fig. 3:**
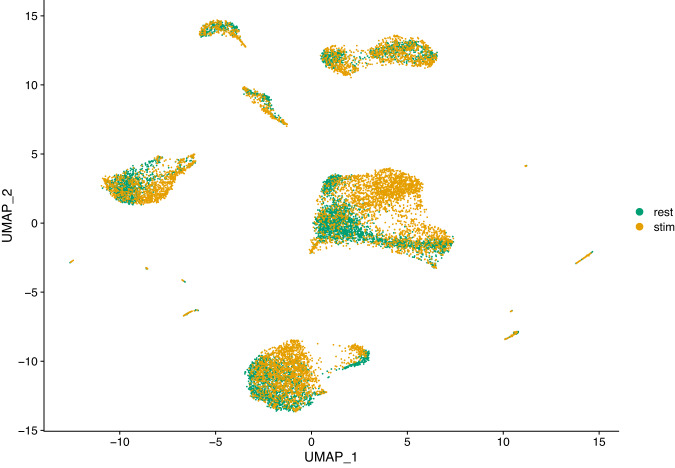
UMAP plot of scRNA-seq data for the resting versus stimulated PBMC cells after integration with the CCA method. rest = resting PBMCs, stim = stimulated PBMCs.

**Fig. 4:**
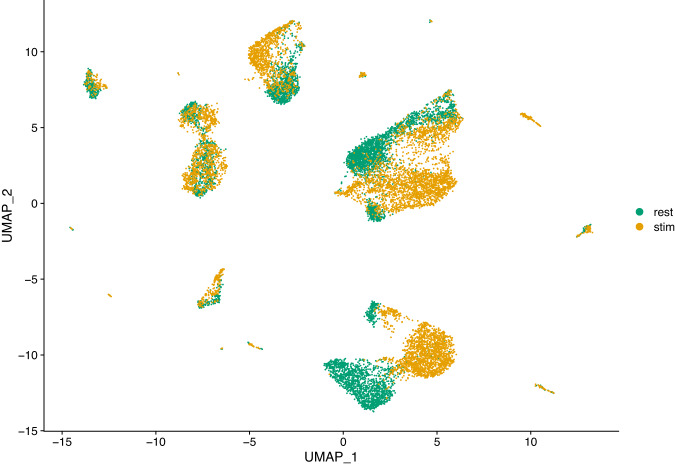
UMAP plot of scRNA-seq data for the resting versus stimulated PBMC cells after integration with the RPCA method. rest = resting PBMCs, stim = stimulated PBMCs.

**Fig. 5:**
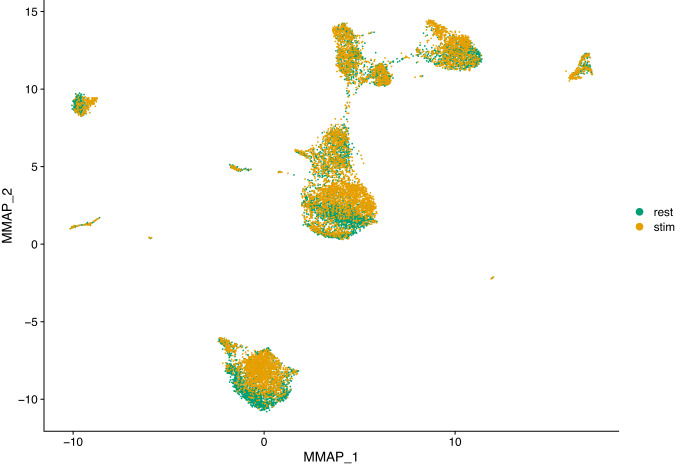
UMAP plot of scRNA-seq data for the resting versus stimulated PBMC cells after integration with the MultiMAP method. rest = resting PBMCs, stim = stimulated PBMCs.

**Fig. 6:**
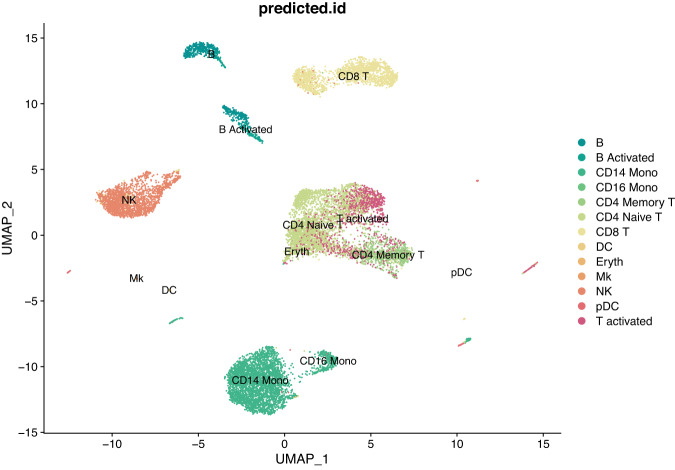
UMAP plot of scRNA-seq data for the resting versus stimulated PBMC cells after integration with the CCA method and clustering.

High-throughput single-cell transcriptomics has become a very popular and powerful tool for unbiased profiling of complex and heterogeneous cellular systems, thanks to the availability of commercialized workflows and improvements in cost and throughput^10^. The main applications of this technology are related to the discovery of cell types and states, the reconstruction of cell trajectories and fate decisions, and the modelling of spatially complex tissues. However for most studies data is generated separately, i.e. at different times and with different operators. For very large scale studies (e.g. a cell atlas), data may also be generated in multiple laboratories, with different cell dissociation protocols. These factors result in batch effects, where the expression of a gene in a batch differs systematically from that in another batch. These differences may introduce spurious structures in the data or mask the underlying biology, leading to wrong conclusions. Thus, it is necessary to correct such effects before further analysis^11^. Several methods have been developed to correct batch effects in single-cell transcriptomics data, using different statistical and mathematical frameworks (see for example^5,7,12,13^). It is essential to benchmark all those methods properly in order to evaluate precisely their respective strengths and weaknesses. For instance, Haghverdi and colleagues^13^ use different datasets to benchmark their mutual nearest-neighbor (MNN) algorithm: simulated data for a simple scenario with two batches of cells having varying proportions of three cell types, two hematopoietic datasets generated in different laboratories using different scRNA-seq protocols (SMART-seq2 and MARS-seq) and a complex dataset on human pancreatic tissue composed of four different sources of data, generated using two different scRNA-seq protocols (SMART-seq2 and CEL-seq2). Obviously, datasets generated from biological samples are highly valuable for these type of benchmarking studies, preferably generated using a variety of scRNA-seq protocols. Hence the interest in our resting versus stimulated PBMC dataset. The 13,263 resting and stimulated cells of our dataset are clearly separated on the two dimensional UMAP projection (Fig. 2). Since the two populations are composed of the same cell types and originate from the same sample, data integration (i.e. batch correction) algorithms should be able to re-align and group the different cell types. To illustrate this, we applied three different methods to our dataset: the Seurat method CCA^12^, the reciprocal PCA method described in the Seurat package (RPCA) and the MultiMAP method implemented in the Python package of the same name^7^. The Seurat alignment workflow (CCA) is based on the canonical correlation analysis, which aims at finding linear combinations of features across datasets that are maximally correlated, thus identifying shared correlation structures. The first step of this method is to find cell pairs that are in a matched biological state (called ‘anchors’) that will be used to correct the differences between datasets. The reciprocal PCA (RPCA) is a slightly modified version of the CCA workflow, using reciprocal PCA to identify the ‘anchors’ between the datasets. This procedure is faster and constitute a more conservative approach where cells in different biological states are less likely to align after integration. The Seurat developers recommend using RPCA when a substantial fraction of the cells in one dataset have no matching type in the other, when datasets originate from the same platform, or when there are a large number of datasets or cells to integrate. MultiMAP^7^ is an algorithm for the dimensionality reduction and integration of multiple datasets. It constructs a nonlinear manifold on which diverse high dimensional data reside and then projects the manifold and data into a shared low-dimensional embedding space. MultiMAP is a generalization of the UMAP algorithm for multiple datasets with different dimensions. It can integrate any number of datasets, can leverage features that are not present in all datasets and is scalable to very large datasets. Figures 3–5 show UMAP graphs of the resting and stimulated datasets after integration with CCA, RPCA and MultiMAP methods respectively. We can see that after integration the cell clusters overlap to a large extend, with no large cluster composed of only one condition. Also visible on the different graphs is that the different methods have different performances. For instance it is clear that the overlap of cells for the two different conditions is better in the case of CCA (Fig. 3) or MultiMAP (Fig. 5) than for RPCA (Fig. 4). After the integration step, we can cluster the cells according to their expression profile. Figure 6 shows the resting and stimulated datasets after integration with CCA and clustering. As expected, the different clusters display homogeneous cell types, indicating good integration performance. Datasets such as the one proposed here have already been used to develop and refine data integration methods and to investigate how human PBMCs vary in response to immune system stimulation^12^. An interesting way to benchmark these data integration methods is to remove abundant or rare cells from one of the conditions (e.g., the stimulated set) and run the alignment procedure to see how it handles the missing data. More than 50 integration methods are currently available for single-cell data integration. In order to efficiently benchmark them, there is a need for an objective methodology and metrics, i.e. the ability of the methods to remove batch effects while preserving biological variation, and of course a diverse set of robust datasets that reflect the variety and complexity of nonlinear, nested batch effects that can occur in real experimental conditions^14^.

**Fig. 7:**
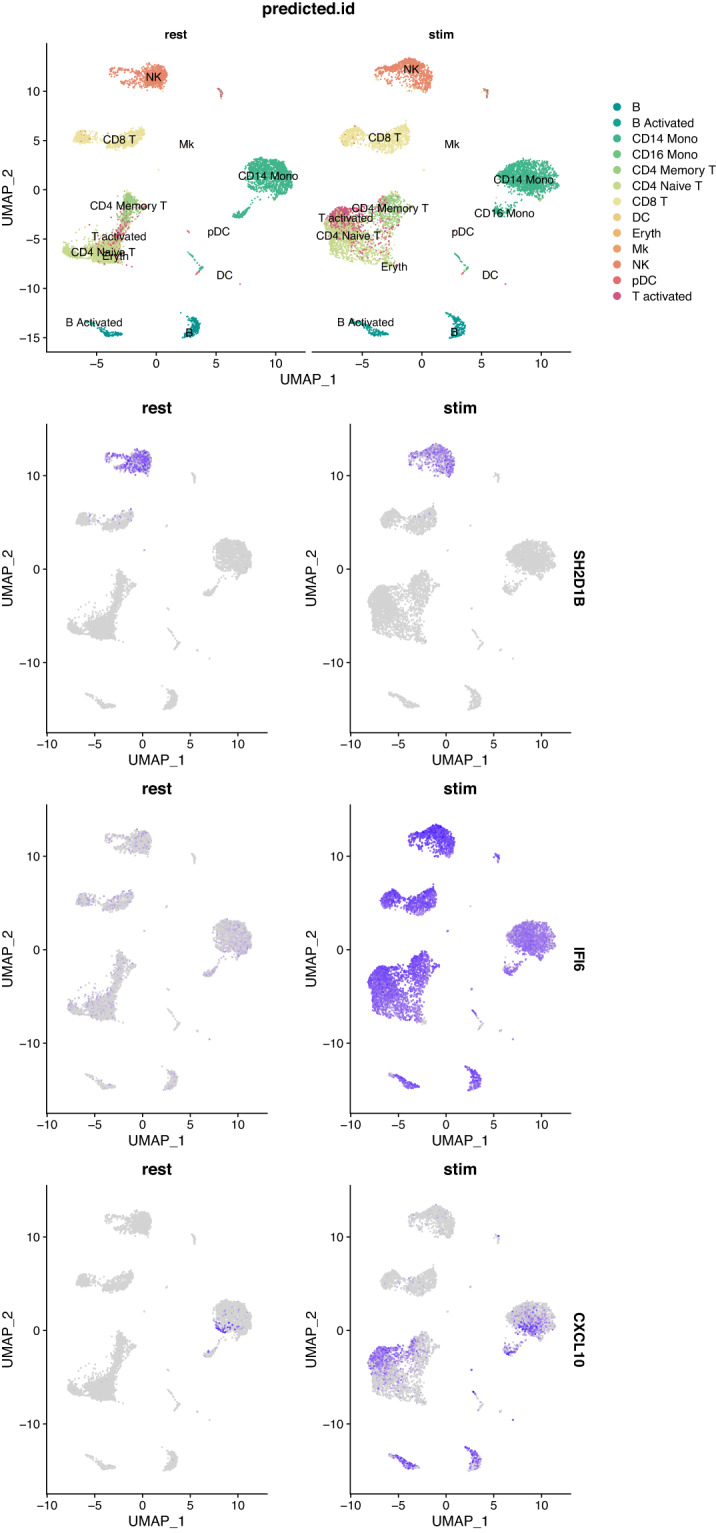
Visualisation of the expression of some genes. The top panel shows resting (left) and stimulated (right) PBMCs labelled by predicted cell identities. The lower panels show the same UMAP projections coloured by gene expression for the genes *SH2D1B*, *IFI6* and *CXCL10*.

Once the cells have been integrated and the different cell types identified, it is possible to look for cell type markers that are conserved in both resting and stimulated conditions, or to compare the datasets to find cell type specific responses to stimulation. Here, we used the “FindConservedMarkers” function of the Seurat R package to identify canonical cell type marker genes that are conserved across the two conditions. This function performs a differential gene expression test for the for each group, in our case resting and stimulated, and combines the p-values using a meta-analysis method^12^. As an example, we calculated the genes that are conserved markers in both stimulated and resting NK (Natural Killer) cells, which are an important component of the innate immune response. We found that 1,088 genes have adjusted p-values for both resting and stimulated groups <0.05, of which 91 also have a fold-change greater than 1. Among these potential conserved marker genes, *SH2D1B* (also known as *EAT2*) is a cytoplasmic adaptor that regulates receptors of the signaling lymphocyte activation molecule (SLAM) family^15^. This gene is known to play a role in regulating the effector functions of natural killer (NK) cells by controlling signal transduction^16^. The Fig. 7 shows that this gene is indeed expressed in both resting and stimulated NK cells, but its expression is restricted to this cell type, thus defining a conserved marker. The contrast is quite striking with the gene *IFI6* (Interferon alpha inducible protein 6^17^), also shown in the Fig. 7, which is a core interferon response gene and is upregulated in all cell types in the stimulated dataset. Using the “FindMarkers” function of the Seurat package, we can find out which genes are differentially expressed in resting and stimulated cells of the same type. For example, for B cells, we find 986 genes that are differentially expressed between resting and stimulated conditions with an adjusted p-value < 0.05. Among them, *CXCL10* is significantly differentially expressed with an adjusted p-value of 1.45 × 10^−81^ and an average log2 fold change of 4.9, and indeed appears strongly upregulated in stimulated B cells on the Fig. 7, but also in monocytes and some T cells. *CXCL10* is a pro-inflammatory cytokine involved in a variety of processes including chemotaxis, differentiation and activation of peripheral immune cells. It plays an important role during viral infections by stimulating the activation and migration of immune cells to the infected sites^18^. Note that more samples would be needed to strengthen the observations that we make here on this dataset.

### Fresh and frozen PBMCs samples

**Fig. 8:**
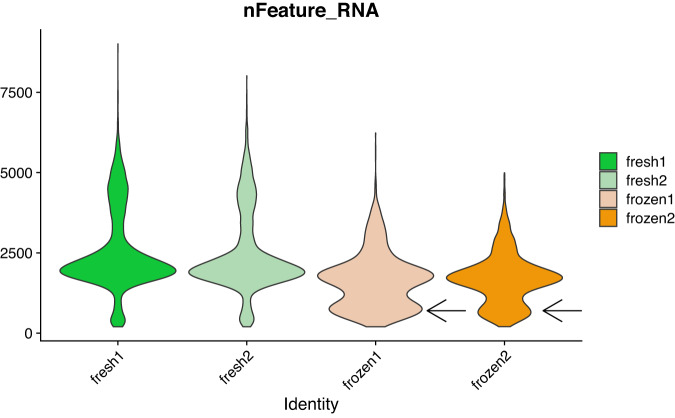
Violin plot of the number of genes per cell for the fresh and frozen samples. The two black arrows indicate anomalies detected for the frozen samples.

**Fig. 9:**
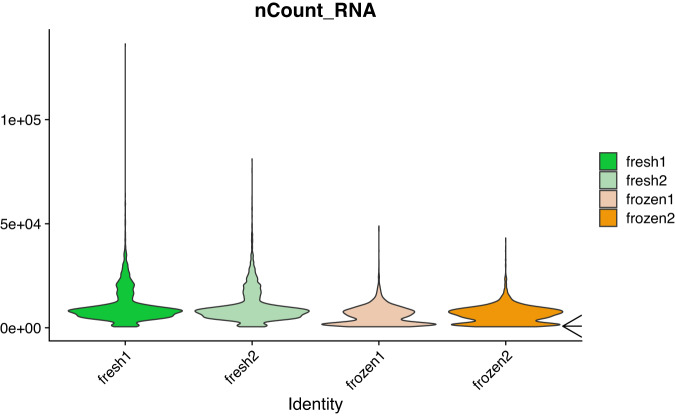
Violin plot of the number of molecules per cell for the fresh and frozen samples. The black arrow indicate an anomaly detected for the frozen samples.

**Fig. 10:**
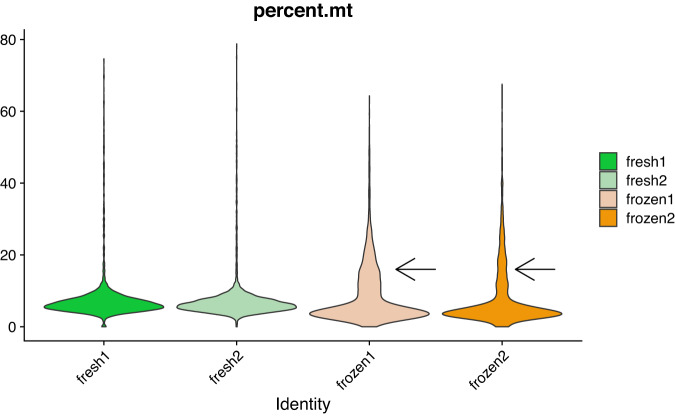
Violin plot of the percentage of mitochondrial genes per cell for the fresh and frozen samples. The black arrow indicate an anomaly detected for the frozen samples.

As described in the methods, we used technical replicates issued from the same individual to avoid biological variations which are quite common for PBMCs. In order to have realistic conditions for the frozen samples, they were kept in the freezer for seven days. For the fresh PBMCs, cells were processed immediately for single-cell library preparation as recommended by the manufacturer and as recommended for best results in single-cell library preparation^19^. It is well known that once tissue samples have been removed from a patient, they promptly begin to undergo gene expression changes and RNA degradation^20,21^. Therefore it was not possible to process fresh and frozen samples the same day and on the same machine for our experimental design. This might theoretically induce a batch effect if the 10X Chromium machine has some technical problem but we think that it is very unlikely. A technical test note from 10X Genomics^22^ shows that for four PBMC libraries generated on three different chips (thus three different dates) the number of genes recovered and the cell clustering are very similar. There is also a critical step during the 10X library preparation, after the run of the chromium controller for the GEMs (Gel Beads-in-emulsion) generation where it is easy to visually assess whether the emulsion is correct, and we did not detect any problem at this step for the two runs. Finally, if a technical problem had occurred at the library step preparation, we would likely see major differences in the quality control values displayed in Table 2, which is not the case. However, it is true that theoretically such a batch effect could occur and therefore it is important to be cautious for the analysis of these samples. In Table 2, we have seen that the median number of detected genes is lower in the frozen samples (around 1600 genes per cell). The distribution of the number of genes detected per cell (Fig. 8) is also slightly different for the frozen samples. We see that there is a higher proportion of cells having a number of detected genes well below the median (black arrows on the figure). In fact, 23 and 32% of the cells in the frozen samples have less than 1,100 detected genes. For the frozen samples(Fig. 9) 27 and 37% of the cells have a molecule count below 3,500. Finally, the percentage of mitochondrial genes (Fig. 10) is high in frozen samples: 24 and 30% of cells have a percentage higher than 10%. All these deviations, that we do not observe for the fresh PBMCs, could be indicative of a relatively high proportion of cells in a state of elevated levels of stress or even dying, probably as a direct consequence of the freeze/thaw protocol. Recently, some groups have tried to develop protocols that enable preservation of cells and storage for later scRNA-seq processing and analysis^23,24^. Some of those protocols use dimethyl sulfoxide (DMSO) or methanol to cryopreserve cells. DMSO is frequently used to preserve animal cells and to prevent the formation of ice crystals. Methanol, on the other hand, dehydrate cells and cause nucleic acids to appear in a collapsed form, thus preserving them with only minor modifications. It is worth noting that some commercial formulations are readily available for scRNA-seq-compatible cell preservation.

## Data Availability

All custom R and python scripts for quality control, data integration, figures and analysis are available on our GitHub repository (https://github.com/erbon7/sc_pbmc).
